# Clinical and pathological features of antinuclear antibody-positive drug-induced liver injury compared with autoimmune hepatitis: A retrospective study

**DOI:** 10.1097/MD.0000000000045750

**Published:** 2025-11-21

**Authors:** Wanting Liu, Zhendong Zhang, Xiaohan Ma, Xiangyu Zeng, Songsong Yuan, Xueqin Li, Feng Nie, Lixia Yang

**Affiliations:** a Department of Infectious Diseases, The First Affiliated Hospital of Nanchang University, Nanchang, China; b Department of Pathology, The First Affiliated Hospital of Nanchang University, Nanchang, China; c Department of Gastroenterology, Wuhan Jinyintan Hospital, Wuhan, China; d Department of Special Needs Medical Ward, Nanxishan Hospital of Guangxi Zhuang Autonomous Region, Guilin, China.

**Keywords:** antinuclear antibody, autoimmune hepatitis, drug-induced liver injury

## Abstract

Drug-induced liver injury (DILI) and autoimmune hepatitis (AIH) often present with overlapping clinical and pathological features, which makes the differential diagnosis particularly challenging in antinuclear antibody-positive (ANA-positive) cases. This study aims to provide new insights into addressing this diagnostic challenge and improving the distinction between ANA-positive DILI and AIH. A total of 87 DILI and 82 AIH cases who underwent liver biopsy at The First Affiliated Hospital of Nanchang University between January 2013 and December 2023 were enrolled. Among them, 42 cases were ANA-positive DILI. A retrospective observational study was conducted on the above patients. We observed that the detection rate of antinuclear antibodies was significantly higher in female patients. Compared to the AIH group, the ANA-positive DILI group exhibited lower levels of the S index, serum globulin, activated partial thromboplastin time, immunoglobulin G, immunoglobulin A, and immunoglobulin M, while presenting higher levels of total bilirubin and blood platelets (all *P* <.05). Both the DILI and AIH groups predominantly exhibited ANA positivity. Notably, the anti-smooth muscle antibody positivity rate was significantly higher in the AIH group compared to the ANA-positive DILI group. The AIH group exhibited more severe liver inflammation and fibrosis compared to the ANA-positive DILI group. Neutrophil infiltration was predominant in the ANA-positive DILI group, whereas lymphocytic and plasma cell infiltration was more pronounced in the AIH group. When DILI patients tested positive for antinuclear antibodies, significant differences in liver function, complete blood count, immunoglobulin levels, and liver histopathological characteristics remained between them and AIH patients. For cases in which a definitive diagnosis is difficult, early liver biopsy and long-term follow-up are recommended to monitor disease progression.

## 1. Introduction

Drug-induced liver injury (DILI) is caused by chemical drugs, biological products, proprietary Chinese medicines, as well as herbal medicines, natural drugs, health products, and dietary supplements. Based on the mechanism of injury, DILI is classified into intrinsic, idiosyncratic, and indirect types.^[1]^ Autoimmune hepatitis (AIH) is a noninflammatory liver disease mediated by abnormal autoimmune responses. AIH is characterized by distinct histological patterns, including interface hepatitis, lymphocytic and plasma cell infiltration and hepatocyte rosette, whereas DILI exhibits diverse patterns of injury.^[2,3]^ Currently, both DILI and AIH lack highly specific and sensitive diagnostic biomarkers, and overlapping histopathological features further complicate differentiation between the 2 conditions.^[4,5]^ When DILI induces liver damage through immune-mediated inflammatory responses, such as idiosyncratic DILI with antinuclear antibody (ANA) positivity, or when AIH cannot exclude potential drug involvement, differentiation becomes even more challenging.^[4]^ Symptoms of DILI typically improve significantly following the discontinuation of the suspected drug and routine symptomatic treatment, whereas AIH requires immunosuppressive therapy to achieve disease remission. If untreated, AIH may progress to liver fibrosis, cirrhosis, or even liver failure. Therefore, early identification of these 2 conditions is critically important.

Although numerous studies have reported comparisons between the laboratory indicators and liver histopathology of DILI and AIH, there is a lack of analyses specifically addressing the differences between ANA-positive DILI and AIH.^[6–8]^

## 2. Methods

To further investigate whether quantifiable laboratory indicators or liver histological differences exist between ANA-positive DILI and AIH patients, this study collected clinical data from 87 DILI and 82 AIH patients who underwent liver biopsy at The First Affiliated Hospital of Nanchang University from January 2013 to December 2023. Among them, 42 cases were ANA-positive DILI and 75 cases were ANA-positive AIH. Demographic data, clinical symptoms and signs, laboratory indicators, and histopathological assessment data were analyzed to provide insights for distinguishing these 2 conditions in clinical practice.

### 2.1. Study population

Evaluate cases of DILI and AIH who underwent liver biopsy at The First Affiliated Hospital of Nanchang University from January 2013 to December 2023. Inclusion criteria: (1) DILI patients met the Roussel-Uclaf Causality Assessment Method score ≥6;^[9]^ (2) AIH patients met the simplified diagnostic criteria for AIH proposed by the International AIH Group (IAIHG) with a score ≥6.^[4]^ All enrolled patients underwent at least 6 months of follow-up observation to confirm diagnostic features. Exclusion criteria: cases with viral hepatitis, alcoholic liver disease, fatty liver, hereditary metabolic liver diseases, autoimmune diseases other than AIH, liver cancer, history of liver transplantation, or incomplete data were excluded.

### 2.2. Data collection

Demographic data, clinical symptoms, and signs: gender, age, and presenting symptoms and signs during the illness.

#### 2.2.1. Laboratory indicators

Noninvasive liver fibrosis model indicators (S index = 1000 × GGT/ (PLT × ALB^2^)), liver function (ALT, AST, TBIL, DBIL, ALB, GLB, GGT, ALP), lipid profile (TC, TG, HDL, LDL), complete blood count (WBC, Hb, PLT), coagulation function (PT, INR, APTT), immunoglobulins (IgG, IgA, IgM), ANA titers (1:100, 1:320, 1:1000), ANA fluorescence patterns (cytoplasmic granular, antinuclear membrane, cytoplasmic fibrous, nuclear homogeneous, nucleolar, nuclear granular, nuclear fimbriated), and types of autoantibodies.

#### 2.2.2. Pathological assessment

All patients underwent percutaneous liver biopsy under ultrasound guidance, with a liver tissue length of ≥ 1.0 cm and at least 6 portal areas evaluable under the microscope. Pathological diagnoses were performed jointly by 2 experienced liver pathologists. All specimens were stained with hematoxylin and eosin, and retrospective collection of liver pathology observations from all enrolled patients was conducted. The degree of liver inflammation and fibrosis was assessed based on the G and S staging system for chronic hepatitis, revised by domestic scholars in 1995 using the Scheuer scoring system.^[10]^

Clinical and laboratory information was retrieved from patients’ medical records. The study protocol was reviewed and approved by the Ethics Committee of The First Affiliated Hospital of Nanchang University (Approval No. CDYFY-IACUC-202501GR104).

### 2.3. Statistical analysis

Data analysis was performed using SPSS 26.0 statistical software (Chicago). Normality tests were conducted on the data. Measures with normal and approximate normal distribution were expressed as mean ± SD, and measures with skewed distribution were expressed as median M (P25, P75). Categorical data are expressed as proportions and rates. Continuous variables were compared using t-tests or Mann-Whitney U tests. Categorical variables were compared using chi-square tests or Fisher exact tests. A statistically significant difference of *P* <.05 was used as the test level.

## 3. Results

### 3.1. General information

Based on the inclusion and exclusion criteria, a total of 169 patients were enrolled in this study, including 87 cases in the DILI group, of which 48 were female (55.2%), with an average age of 45.25 ± 13.878 years, and 82 cases in the AIH group, of which 71 were female (86.6%), with an average age of 48.85 ± 12.375 years. In terms of gender, the proportion of female patients in the AIH group was significantly higher than that in the DILI group (*P* <.05). Both groups were predominantly composed of middle-aged patients, and there was no statistically significant difference in age between the 2 groups (*P* >.05). No statistically significant differences were observed in gender or age between the ANA-positive DILI group and the AIH group (*P* >.05).

### 3.2. Clinical symptoms

In terms of clinical symptoms, the incidence of skin itching, fatigue, poor appetite, nausea and vomiting, discomfort in the liver area, and jaundice in the DILI group was higher than that in the AIH group, with statistically significant differences observed for poor appetite and nausea and vomiting between the 2 groups (*P* <.05) (Table 1). When comparing the ANA-positive DILI group and the AIH group, significant differences were also found for poor appetite and nausea and vomiting (*P* <.05) (Table 2).

**Table 1:** Comparison of clinical symptoms between the DILI group and the AIH group.

Clinical symptoms	DILI group (n = 87)	AIH group (n = 82)	Statistical value	*P*-value
Skin itching	15 (17.2)	12 (14.6)	χ² = 0.214	.64
Fatigue	52 (59.8)	41 (50.0)	χ² = 1.628	.20
Poor appetite	46 (52.9)	23 (28.0)	χ² = 10.768	<.001
Nausea and vomiting	31 (35.6)	14 (17.1)	χ² = 7.442	.006
Discomfort in liver area	32 (36.8)	25 (30.5)	χ² = 0.748	.39
Jaundice	43 (49.4)	40 (48.8)	χ² = 0.007	.93

AIH = autoimmune hepatitis, DILI = drug-induced liver injury.

**Table 2:** Comparison of clinical symptoms between the ANA-positive DILI group and the AIH group.

Clinical symptoms	ANA-positive DILI group (n = 42)	AIH group (n = 82)	Statistical value	*P*-value
Skin itching	7 (16.7)	12 (14.6)	χ² = 0.088	.77
Fatigue	25 (59.5)	41 (50.0)	χ² = 1.012	.31
Poor appetite	20 (47.6)	23 (28.0)	χ² = 4.696	.03
Nausea and vomiting	14 (33.3)	14 (17.1)	χ² = 4.201	.04
Discomfort in liver area	14 (33.3)	25 (30.5)	χ² = 0.104	.75
Jaundice	21 (50.0)	40 (48.8)	χ² = 0.017	.90

AIH = autoimmune hepatitis, ANA = antinuclear antibody, DILI = drug-induced liver injury.

### 3.3. Laboratory indices

In terms of the serum noninvasive diagnostic model for liver fibrosis, the S index in the DILI group was lower than that in the AIH group. Regarding liver function, the ALB level in the DILI group was higher than that in the AIH group, while the GLB level in the DILI group was lower than that in the AIH group. In terms of lipid profile, both TC and LDL levels in the DILI group were higher than those in the AIH group. For complete blood count, both Hb and PLT levels in the DILI group were higher than those in the AIH group. Regarding coagulation function, PT, INR, and APTT in the DILI group were all lower than in the AIH group. In terms of immune-related factors, IgG, IgA, IgM, and ANA titers in the DILI group were all lower than those in the AIH group, and in the ANA fluorescence patterns, the proportions of cytoplasmic granular type and nuclear granular type in the DILI group were both lower than those in the AIH group. The differences in the above indicators were statistically significant (*P* <.05) (Table 3). The S index in the ANA-positive DILI group was lower than that in the AIH group. Regarding liver function, the TBIL level in the ANA-positive DILI group was higher than that in the AIH group, while the GLB level in the ANA-positive DILI group was lower than that in the AIH group. In terms of complete blood count, the PLT in the ANA-positive DILI group was higher than that in the AIH group. For coagulation function, APTT in the ANA-positive DILI group was lower than that in the AIH group. In terms of immune-related factors, IgG, IgA, and IgM levels in the ANA-positive DILI group were all lower than those in the AIH group. The differences in the above indicators were statistically significant (*P* <.05) (Table 4).

**Table 3:** Comparison of laboratory indices between the DILI group and the AIH group.

Laboratory indices	DILI group (n = 87)	AIH group (n = 82)	Statistical value	*P*-value
S index	0.44 (0.22–0.98)	0.82 (0.37–1.78)	Z = −2.627	.009
ALT	158.45 (62.50–471.53)	157.00 (74.75–425.75)	Z = −0.407	.68
AST	136.65 (48.88–418.68)	161.00 (81.75–396.38)	Z = −0.832	.41
TBIL	33.45 (17.75–149.70)	30.80 (15.13–87.78)	Z = −1.598	.11
DBIL	13.55 (4.20–98.35)	17.05 (4.10–63.10)	Z = −1.096	.27
ALB	39.20 ± 6.67	36.25 ± 5.36	*t* = 3.162	.002
GLB	26.45 (23.50–30.88)	34.25 (29.25–39.10)	Z = −6.508	<.001
GGT	133.50 (50.00–251.25)	153.50 (94.95–253.00)	Z = −0.931	.35
ALP	152.50 (113.28–238.38)	161.45 (109.80–233.50)	Z = −0.093	.93
TC	4.33 (3.64–5.09)	3.91 (3.26–4.65)	Z = −2.326	.02
TG	1.45 (1.05–2.38)	1.57 (1.08–2.13)	Z = −0.171	.86
HDL	1.04 (0.29–1.37)	0.84 (0.44–1.27)	Z = −0.396	.69
LDL	2.41 (1.86–3.14)	1.97 (1.55–2.81)	Z = −2.356	.02
WBC	5.20 (4.06–6.40)	4.97 (3.90–6.15)	Z = −0.557	.58
Hb	126.50 (112.75–139.25)	120.00 (108.00–127.25)	Z = −2.791	.005
PLT	197.00 (148.00–313.00)	168.00 (116.75–231.75)	Z = −2.307	.02
PT	11.90 (11.00–13.13)	12.55 (11.50–14.13)	Z = −3.091	.002
INR	1.01 (0.93–1.11)	1.07 (1.01–1.22)	Z = −3.587	<.001
APTT	29.20 (26.15–34.65)	32.70 (28.30–37.03)	Z = −3.581	<.001
IgG	11.85 (10.45–13.80)	18.55 (14.35–23.42)	Z = −7.722	<.001
IgA	2.25 (1.64–2.90)	3.05 (2.22–3.89)	Z = −3.566	<.001
IgM	1.07 (0.77–1.47)	1.60 (1.05–2.45)	Z = −4.214	<.001
ANA titers				
0	45 (51.7)	7 (8.5)	Z = −5.946	<.001
1:100	27 (31.0)	37 (45.1)		
1:320	14 (16.1)	34 (41.5)		
1:1000	1 (1.1)	4 (4.9)		
ANA fluorescence patterns				
Cytoplasmic granular	13 (14.9)	36 (43.9)	χ2 = 17.196	<.001
Antinuclear membrane	0 (0.0)	4 (4.9)	χ2 = 2.492	.11
Cytoplasmic fibrous	3 (3.4)	3 (3.7)	χ2 ≤0.001	>.99
Nuclear homogeneous	11 (12.6)	17 (20.7)	χ2 = 1.998	.16
Nucleolar	3 (3.4)	3 (3.7)	χ2 ≤0.001	>.99
Nuclear granular	17 (19.5)	35 (42.7)	χ2 = 10.613	<.001
Nuclear fimbriated	1 (1.1)	4 (4.9)	χ2 = 0.952	.33

AIH = autoimmune hepatitis, ALB = albumin, ALP = alkaline phosphatase, ALT = alanine aminotransferase, APTT = activated partial thromboplastin time, AST = aspartate aminotransferase, DBIL = direct bilirubin, DILI = drug-induced liver injury, GGT = gamma glutamyl transferase, GLB = serum globulin, Hb = hemoglobin, HDL = high-density lipoprotein, IgA = immunoglobulin A, IgG = immunoglobulin G, IgM = immunoglobulin M, INR = international normalized ratio, LDL = low density lipoprotein, PLT = blood platelets, PT = prothrombin time, TBIL = total bilirubin, TC = total cholesterol, TG = triglyceride, WBC = white blood cell.

**Table 4:** Comparison of laboratory indices between the ANA-positive DILI group and the AIH group.

Laboratory indices	ANA-positive DILI group (n = 42)	AIH group (n = 82)	Statistical value	*P*-value
S index	0.38 (0.22–0.79)	0.82 (0.37–1.78)	Z = −2.017	.04
ALT	196.00 (64.70–532.20)	157.00 (74.75–425.75)	Z = −0.937	.35
AST	219.00 (61.50–483.70)	161.00 (81.75–396.38)	Z = −0.689	.49
TBIL	36.80 (19.20–158.10)	30.80 (15.13–87.78)	Z = −2.125	.03
DBIL	21.70 (5.30–110.20)	17.05 (4.10–63.10)	Z = −1.769	.08
ALB	37.46 ± 6.77	36.25 ± 5.36	*t* = 1.082	.28
GLB	27.10 (22.90–32.40)	34.25 (29.25–39.10)	Z = −4.736	<.001
GGT	124.00 (68.20–220.00)	153.50 (94.95–253.00)	Z = −0.750	.45
ALP	142.00 (105.00–185.40)	161.45 (109.80–233.50)	Z = −0.177	.86
TC	4.25 (3.10–5.10)	3.91 (3.26–4.65)	Z = −1.075	.28
TG	1.53 (0.90–2.35)	1.57 (1.08–2.13)	Z = −0.288	.77
HDL	0.99 (0.25–1.31)	0.84 (0.44–1.27)	Z = −0.536	.59
LDL	2.48 (1.80–3.21)	1.97 (1.55–2.81)	Z = −1.460	.14
WBC	4.89 (3.61–5.99)	4.97 (3.90–6.15)	Z = −0.539	.59
Hb	124.00 (112.00–133.00)	120.00 (108.00–127.25)	Z = −1.447	.15
PLT	204.00 (178.00–255.00)	168.00 (116.75–231.75)	Z = −2.270	.02
PT	12.20 (11.30–13.70)	12.55 (11.50–14.13)	Z = −0.792	.43
INR	1.03 (0.96–1.19)	1.07 (1.01–1.22)	Z = −0.988	.32
APTT	29.50 (27.20–35.10)	32.70 (28.30–37.03)	Z = −2.236	.03
IgG	12.10 (10.50–14.50)	18.55 (14.35–23.42)	Z = −5.611	<.001
IgA	2.18 (1.62–3.41)	3.05 (2.22–3.89)	Z = −2.232	.03
IgM	1.16 (0.90–1.53)	1.60 (1.05–2.45)	Z = −2.887	.004
ANA titers				
0	0 (0.0)	7 (8.5)	Z = −0.586	.56
1:100	27 (64.3)	37 (45.1)		
1:320	14 (33.3)	34 (41.5)		
1:1000	1 (2.4)	4 (4.9)		
ANA fluorescence patterns				
Cytoplasmic granular	13 (31.0)	36 (43.9)	χ2 = 1.949	.16
Antinuclear membrane	0 (0.0)	4 (4.9)	χ2 = 0.843	.36
Cytoplasmic fibrous	3 (7.1)	3 (3.7)	χ2 = 0.171	.68
Nuclear homogeneous	11 (26.2)	17 (20.7)	χ2 = 0.473	.49
Nucleolar	3 (7.1)	3 (3.7)	χ2 = 0.171	.68
Nuclear granular	17 (40.5)	35 (42.7)	χ2 = 0.056	.81
Nuclear fimbriated	1 (2.4)	4 (4.9)	χ2 = 0.035	.85

AIH = autoimmune hepatitis, ALB = albumin, ALP = alkaline phosphatase, ALT = alanine aminotransferase, ANA = antinuclear antibody, APTT = activated partial thromboplastin time, AST = aspartate aminotransferase, DBIL = direct bilirubin, DILI = drug-induced liver injury, GGT = gamma glutamyl transferase, GLB = serum globulin, Hb = hemoglobin, HDL = high-density lipoprotein, IgA = immunoglobulin A, IgG = immunoglobulin G, IgM = immunoglobulin M, INR = international normalized ratio, LDL = low density lipoprotein, PLT = blood platelets, PT = prothrombin time, TBIL = total bilirubin, TC = total cholesterol, TG = triglyceride, WBC = white blood cell.

In terms of autoantibody types, both the DILI and AIH groups were predominantly ANA-positive, with rates of 42 (48.28%) and 75 (91.46%), respectively. The positive rate of anti-smooth muscle antibody (SMA) (19.51%) in the AIH group was significantly higher than that in the ANA-positive DILI group (4.76%). Moreover, the variety of positive autoantibodies in the AIH group was greater than that in the DILI group (Table 5).

**Table 5:** Comparison of autoantibody types between the DILI group and the AIH group.

Autoantibody types	ANA	AMA-M2	Histone	ANuA	CENP-B	SSA	SSB	Jo-1
DILI group (n = 87)	42 (48.28)	2 (2.30)	1 (1.15)	0 (0.00)	0 (0.00)	4 (4.60)	1 (1.15)	0 (0.00)
AIH group (n = 82)	75 (91.46)	2 (2.44)	3 (3.66)	3 (3.66)	5 (6.10)	14 (17.07)	4 (4.88)	1 (1.22)

Autoantibody types	Ro-52	RNP	PCNA	PM-SCL	Rib-P	Scl-70	Sm	SLA
DILI group (n = 87)	1 (1.15)	1 (1.15)	1 (1.15)	2 (2.30)	0 (0.00)	0 (0.00)	0 (0.00)	0 (0.00)
AIH group (n = 82)	11 (13.41)	4 (4.88)	2 (2.44)	1 (1.22)	1 (1.22)	1 (1.22)	1 (1.22)	1 (1.22)

Autoantibody types	Lc-1	LKM-1	ARA	PCA	ASA	SMA	RNPA	
DILI group (n = 87)	1 (1.15)	0 (0.00)	0 (0.00)	3 (3.45)	0 (0.00)	2 (2.30)	1 (1.15)	
AIH group (n = 82)	1 (1.22)	3 (3.66)	1 (1.22)	3 (3.66)	1 (1.22)	16 (19.51)	0 (0.00)	

AIH = autoimmune hepatitis, AMA-M2 = antimitochondrial antibody M2 subtype, ANA = antinuclear antibody, ANuA = antinuclear antigen antibody, ARA = antiribonuclear antibody, ASA = anti-smooth muscle antibody, CENP-B = centromere protein B, DILI = drug-induced liver injury, Lc-1 = liver cytosol type 1 antibody, LKM-1 = liver kidney microsome type 1 antibody, PCA = parietal cell antibody, PCNA = proliferating cell nuclear antigen, PM-SCL = polymyositis-scleroderma antibody, Rib-P = ribosomal P protein antibody, RNPA = ribonucleoprotein antibody, Scl-70 = topoisomerase I antibody, SLA = soluble liver antigen, Sm = Smith antibody, SMA = smooth muscle antibody, SSA = Sjögren’s syndrome-related antigen A, SSB = Sjögren’s syndrome-related antigen B.

### 3.4. Pathologic features

**Figure 1.**
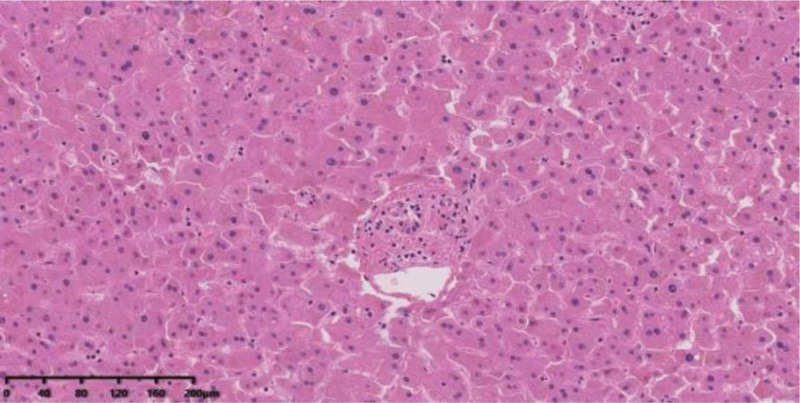
Normal hepatitis. Liver tissue shows no obvious abnormal changes.

**Figure 2.**
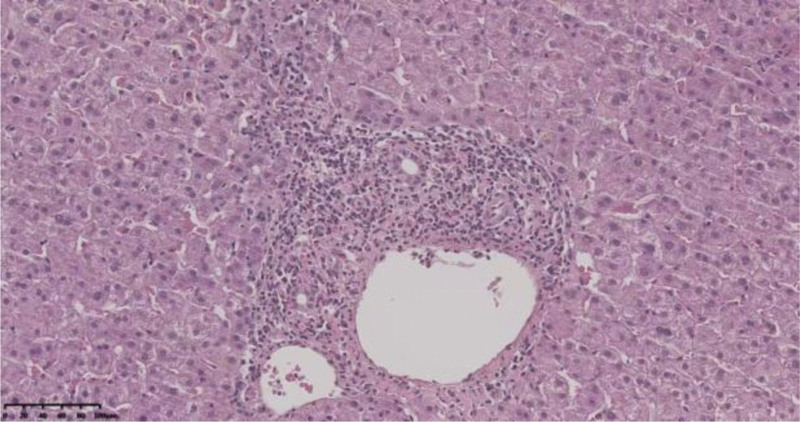
Drug-induced liver injury. Mixed inflammatory cell infiltration is present in the confluent area, with mild interface hepatitis and hyperplasia of the surrounding fine bile ducts.

**Figure 3.**
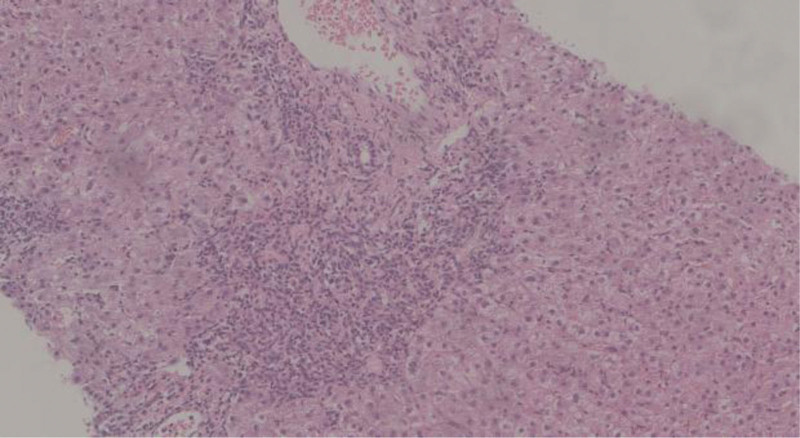
Autoimmune hepatitis. A large lymphocytic and plasma cell infiltration is observed in the confluent area, with severe interface hepatitis and hepatocyte rosette formation. No abnormal changes are seen in the fine bile ducts.

**Figure 4.**
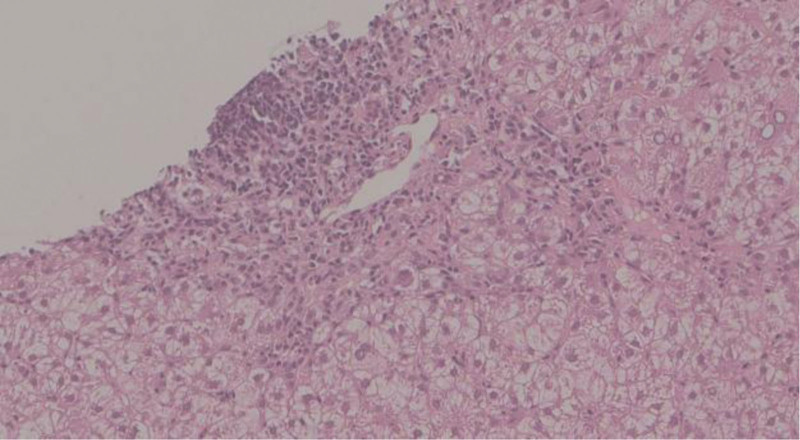
Antinuclear antibody-positive drug-induced liver injury. Multiple mixed inflammatory cell infiltrations are present in the confluent area. Plasma cell interface hepatitis is seen in the confluent area. Liver tissue shows mild to moderate lobular inflammation, and foci of necrosis are observed in hepatocytes surrounding the central vein (arrow). ANA = antinuclear antibody, CV = central vein.

In terms of liver pathology, the DILI group exhibited lower rates of lymphocytic infiltration and plasma cell infiltration compared to the AIH group, while the rates of neutrophil infiltration was higher in the DILI group than in the AIH group. The incidence of hepatocyte rosette in the DILI group was lower than that in the AIH group, whereas the incidence of microgranulomas was higher in the DILI group. The liver inflammation and fibrosis in the DILI group predominantly presented as G1, G2, and S0, S1, while the AIH group predominantly exhibited G2, G3, and S1, S2. The DILI group primarily showed no to mild interface hepatitis, whereas the AIH group displayed mild to moderate interface hepatitis. The differences in the above indicators were statistically significant (*P* <.05). Both groups predominantly showed no to mild lobular inflammation, with no statistically significant difference (*P* >.05) (Table 6). When comparing the ANA-positive DILI group with the AIH group, it was found that the ANA-positive DILI group also had lower rates of lymphocytic infiltration and plasma cell infiltration, and higher rates of neutrophil infiltration infiltration compared to the AIH group. The incidence of hepatocyte rosette in the ANA-positive DILI group was also lower than that in the AIH group, while the incidence of microgranulomas was higher in the ANA-positive DILI group. The liver inflammation and fibrosis in the ANA-positive DILI group predominantly presented as G1, G2, and S0, S1, whereas the AIH group predominantly exhibited G2, G3, and S1, S2. The ANA-positive DILI group primarily showed no to mild interface hepatitis, while the AIH group displayed mild to moderate interface hepatitis. The differences in the above indicators were statistically significant (*P* <.05) (Table 7). Histologic pictures of representative cases are shown (Figs. 1–4).

**Table 6:** Comparison of pathologic features between the DILI group and the AIH group.

Pathologic features	DILI group (n = 87)	AIH group (n = 82)	Statistical value	*P*-value
Inflflammatory cell infiltration				
Lymphocytic	28 (32.2)	62 (75.6)	χ2 = 31.978	<.001
Plasma cell	24 (27.6)	66 (80.5)	χ2 = 47.456	<.001
Eosinophilic	22 (25.3)	12 (14.6)	χ2 = 2.981	.08
Neutrophil	22 (25.3)	10 (12.2)	χ2 = 4.714	.03
Hepatocyte rosette	9 (10.3)	32 (39.0)	χ2 = 18.896	<.001
Hepatocellular edema	53 (60.9)	58 (70.7)	χ2 = 1.803	.18
Microgranulomas	21 (24.1)	9 (11.0)	χ2 = 5.009	.03
Staging of inflammation				
G0	4 (4.6)	0 (0.0)	Z = −4.930	<.001
G1	35 (40.2)	17 (20.7)		
G2	36 (41.4)	27 (32.9)		
G3	12 (13.8)	25 (30.5)		
G4	0 (0.0)	13 (15.9)		
Staging of fibrosis				
S0	30 (34.5)	8 (9.8)	Z = −4.855	<.001
S1	35 (40.2)	27 (32.9)		
S2	11 (12.6)	16 (19.5)		
S3	6 (6.9)	17 (20.7)		
S4	5 (5.7)	14 (17.1)		
Interface hepatitis				
Not have	29 (33.3)	12 (14.6)	Z = −5.017	<.001
Mild	41 (47.1)	22 (26.8)		
Moderate	15 (17.2)	36 (43.9)		
Severe	2 (2.3)	12 (14.6)		
Lobular inflammation				
Not have	73 (83.9)	63 (76.8)	Z = −1.141	.25
Mild	11 (12.6)	15 (18.3)		
Moderate	2 (2.3)	4 (4.9)		
Severe	1 (1.1)	0 (0.0)		

AIH = autoimmune hepatitis, DILI = drug-induced liver injury.

**Table 7:** Comparison of pathologic features between the ANA-positive DILI group and the AIH group.

Pathologic features	ANA-positive DILI group (n = 42)	AIH group (n = 82)	Statistical value	*P*-value
Inflflammatory cell infiltration				
Lymphocytic	12 (28.6)	62 (75.6)	χ 2 = 25.538	<.001
Plasma cell	12 (28.6)	66 (80.5)	χ 2 = 32.081	<.001
Eosinophilic	12 (28.6)	12 (14.6)	χ 2 = 3.456	.06
Neutrophil	11 (26.2)	10 (12.2)	χ 2 = 3.867	.05
Hepatocyte rosette	6 (14.3)	32 (39.0)	χ 2 = 7.998	.005
Hepatocellular edema	26 (61.9)	58 (70.7)	χ 2 = 0.990	.32
Microgranulomas	15 (35.7)	9 (11.0)	χ 2 = 10.890	<.001
Staging of inflammation				
G0	0 (0.0)	0 (0.0)	Z = −3.710	<.001
G1	20 (47.6)	17 (20.7)		
G2	14 (33.3)	27 (32.9)		
G3	8 (19.0)	25 (30.5)		
G4	0 (0.0)	13 (15.9)		
Staging of fibrosis				
S0	16 (38.1)	8 (9.8)	Z = −3.689	<.001
S1	14 (33.3)	27 (32.9)		
S2	5 (11.9)	16 (19.5)		
S3	4 (9.5)	17 (20.7)		
S4	3 (7.1)	14 (17.1)		
Interface hepatitis				
Not have	11 (26.2)	12 (14.6)	Z = −3.495	<.001
Mild	21 (50.0)	22 (26.8)		
Moderate	9 (21.4)	36 (43.9)		
Severe	1 (2.4)	12 (14.6)		
Lobular inflammation				
Not have	33 (78.6)	63 (76.8)	Z = −0.196	.85
Mild	7 (16.7)	15 (18.3)		
Moderate	1 (2.4)	4 (4.9)		
Severe	1 (2.4)	0 (0.0)		

AIH = autoimmune hepatitis, ANA = antinuclear antibody, DILI = drug-induced liver injury.

## 4. Discussion

This study demonstrated that although ANA positivity occurs in both AIH and DILI, there are still important differences between the 2 conditions. Specifically, AIH patients showed significantly elevated immunoglobulin levels (IgG, IgA, IgM) and globulin, whereas ANA-positive DILI patients exhibited higher platelet counts and total bilirubin. Furthermore, the positivity rate of SMA was significantly higher in AIH. Histologically, ANA-positive DILI was distinguished by predominant neutrophil infiltration and microgranulomas, while AIH was characterized by lymphoplasmacytic infiltration, interface hepatitis, hepatocyte rosettes, and more advanced inflammation and fibrosis, although lobular inflammation was more prominent in DILI.

ANA are commonly found in AIH, particularly in Type I AIH. According to the simplified diagnostic criteria proposed by the IAIHG, ANA is one of the main serological markers for AIH.^[4]^ However, the specificity of ANA is relatively low, as it can be detected in various immune-related conditions. When both ANA and SMA are present, the diagnostic accuracy for AIH increases from approximately 58% to 74%, although the coexistence of these 2 autoantibodies is uncommon in clinical practice.^[5]^

When patients with DILI test positive for ANA, this may suggest that the mechanism of liver injury involves immune-mediated injury, where drugs or their metabolites may act as haptens, binding with relevant antibodies and triggering an autoimmune response that leads to ANA production.^[11]^ Thus, ANA positivity may reflect an abnormal immune response to drugs or their metabolites. When patients have an unrecognized genetic susceptibility to autoimmune diseases, drugs can act as “triggering factors” for ANA production.^[12]^

Multiple studies have shown that the prevalence of DILI does not differ between males and females, whereas AIH predominantly affects females, particularly postmenopausal women,^[13–15]^ consistent with the findings of this study. In this study, the detection rate of ANA in female DILI patients was higher than that in males, and there was no statistically significant difference in gender between the ANA-positive DILI and AIH groups, indicating that female patients have a higher likelihood of presenting with ANA positivity in both DILI and AIH. Regarding clinical manifestations, DILI patients exhibited more pronounced symptoms than AIH patients, regardless of ANA status, with poor appetite and nausea/vomiting serving as primary distinguishing features. Given that DILI typically presents acutely and is a leading cause of acute liver failure in some Western countries,^[16]^ it is more likely to result in severe clinical symptoms.

In laboratory indicators, regardless of ANA positivity, DILI patients had lower S index, GLB, APTT, IgG, IgA, and IgM levels compared to AIH patients, while PLT levels were higher in the DILI group. When DILI patients were ANA-positive, there were no statistically significant differences in ALB, TC, LDL, Hb, PT, INR, ANA titers, and ANA fluorescence patterns compared to AIH patients, suggesting that the differences between the 2 diseases are diminished, complicating differential diagnosis. ALB, PT, and INR levels reflect liver synthetic function and are also prognostic indicators of liver disease.^[17]^ The significant statistical differences in ALB, PT, and INR between the DILI and AIH groups suggest a potentially worse prognosis for AIH compared to DILI. However, these differences disappeared when comparing the ANA-positive DILI group with the AIH group, indicating that ANA positivity in DILI patients is associated with a poorer prognosis.^[18]^ Elevated serum IgG and GLB levels are important markers of seropositive AIH.^[5]^ In this study, significant statistical differences were observed in IgG and GLB levels between the AIH group and the ANA-positive DILI group. Some studies suggest that an ANA titer of ≥ 1:320 aids in the early identification of AIH, and higher titers are more diagnostic.^[19]^ However, in the samples included in this study, patients with high ANA titers were relatively rare in both the DILI and AIH groups. Regarding autoantibody types, our findings are consistent with a previous prospective study,^[20]^ showing that the positive rate of SMA in AIH is higher than that in DILI with autoimmune characteristics. However, another validation study did not reach the same conclusion,^[21]^ indicating that the role of classical antibodies in differential diagnosis remains to be discussed in the future. Furthermore, while the specificity of anti-soluble liver antigen antibody (SLA) for AIH diagnosis is significantly higher than that of ANA, only 1 positive case was found in the AIH group, with none in the DILI group.

In this study, regardless of ANA positivity, histological assessments indicated that liver inflammation and fibrosis were generally more severe in AIH patients compared to DILI patients. DILI tends to have an acute onset and is generally less likely to result in liver fibrosis.^[22]^ DILI patients primarily exhibited neutrophil infiltration, while AIH patients showed predominant lymphocytic and plasma cell infiltration. Hepatocellular DILI involves significant portal inflammation, necrosis, and apoptosis, typically affecting zone 3, whereas cholestatic DILI is characterized by small bile duct and hepatocyte cholestasis.^[23]^ DILI often presents with eosinophilic and neutrophil infiltration, exhibiting microgranulomas, generally with lower degrees of liver inflammation and fibrosis, primarily presenting as lobular inflammation.^[24]^ AIH typically exhibits interface hepatitis, lymphocytic and plasma cell infiltration and hepatocyte rosette, although its specificity is not high, with a greater degree of liver inflammation and fibrosis compared to DILI.^[6]^ Additionally, over time, some DILI cases with autoimmune features may progress to AIH,^[25,26]^ and the histological differences between the 2 may diminish in later stages of the disease, highlighting the importance of early liver biopsy. For cases with unclear clinical diagnoses, such as those with ANA positivity and vague drug exposure histories, liver biopsy can not only assist in diagnosis but also provide prognostic information, with a recommendation for long-term follow-up.^[27,28]^ Furthermore, liver biopsy may be more sensitive than transient elastography for assessing liver fibrosis.^[29]^

Identifying ANA-positive DILI patients is crucial for treatment decision-making. DILI typically resolves with the discontinuation of the suspected drug and symptomatic supportive care, effectively preventing ongoing liver damage. Although the use of corticosteroids in DILI treatment remains controversial, a small subset of moderate/severe cases may require glucocorticoid therapy.^[3,11]^ Incorrectly recognizing ANA-positive DILI as AIH and thus directly using immunosuppressive therapy can have unpredictable consequences.

This study is a single-center retrospective analysis, and the enrolled patients all underwent liver biopsy. The small sample size may lead to selection bias. Since this study is cross-sectional in nature, it did not dynamically monitor changes in biochemical, immunological, and pathological features at different stages in patients, as well as their responses to medications and clinical follow-up.

## 5. Conclusion

In conclusion, the distinctions between DILI and AIH are lessened but not eliminated by ANA positivity. Histopathological characteristics, immunoglobulin levels, and SMA positivity remain important discriminators between the 2 conditions. Clinicians should integrate serological, histological, and clinical evidence rather than relying solely on ANA for diagnosis. In cases where the diagnosis is unclear, early liver biopsy and long-term monitoring are recommended to avoid misclassification and inappropriate immunosuppressive treatment.

## Acknowledgments

The authors would like to thank all the doctors, nurses and patients, who contributed to this study.

## Author contributions

**Conceptualization:** Lixia Yang.

**Data curation:** Wanting Liu.

**Formal analysis:** Xiaohan Ma.

**Investigation:** Xiangyu Zeng.

**Resources:** Zhendong Zhang, Xueqin Li.

**Software:** Feng Nie.

**Visualization:** Songsong Yuan.
